# Disturbed Ca^2+^ Homeostasis Increases Glutaminyl Cyclase Expression; Connecting Two Early Pathogenic Events in Alzheimer’s Disease In Vitro

**DOI:** 10.1371/journal.pone.0044674

**Published:** 2012-09-07

**Authors:** Line De Kimpe, Anna Bennis, Rob Zwart, Elise S. van Haastert, Jeroen J. M. Hoozemans, Wiep Scheper

**Affiliations:** 1 Department of Genome Analysis, Academic Medical Center/University of Amsterdam, Amsterdam, The Netherlands; 2 Department of Neurology, Academic Medical Center/University of Amsterdam, Amsterdam, The Netherlands; 3 Department of Pathology, VU University Medical Center, Amsterdam, The Netherlands; Thomas Jefferson University, United States of Amercia

## Abstract

A major neuropathological hallmark of Alzheimer’s disease (AD) is the deposition of aggregated β amyloid (Aβ) peptide in the senile plaques. Aβ is a peptide of 38–43 amino acids and its accumulation and aggregation plays a key role early in the disease. A large fraction of β amyloid is N-terminally truncated rendering a glutamine that can subsequently be cyclized into pyroglutamate (pE). This makes the peptide more resistant to proteases, more prone to aggregation and increases its neurotoxicity. The enzyme glutaminyl cyclase (QC) catalyzes this conversion of glutamine to pE. In brains of AD patients, the expression of QC is increased in the earliest stages of pathology, which may be an important event in the pathogenesis. In this study we aimed to investigate the regulatory mechanism underlying the upregulation of QC expression in AD. Using differentiated SK-N-SH as a neuronal cell model, we found that neither the presence of Aβ peptides nor the unfolded protein response, two early events in AD, leads to increased QC levels. In contrast, we demonstrated increased QC mRNA levels and enzyme activity in response to another pathogenic factor in AD, perturbed intracellular Ca^2+^ homeostasis. The QC promoter contains a putative binding site for the Ca^2+^ dependent transcription factors c-fos and c-jun. C-fos and c-jun are induced by the same Ca^2+^-related stimuli as QC and their upregulation precedes QC expression. We show that in the human brain QC is predominantly expressed by neurons. Interestingly, the Ca^2+^- dependent regulation of both c-fos and QC is not observed in non-neuronal cells. Our results indicate that perturbed Ca^2+^ homeostasis results in upregulation of QC selectively in neuronal cells via Ca^2+^- dependent transcription factors. This suggests that disruption of Ca^2+^ homeostasis may contribute to the formation of the neurotoxic pE Aβ peptides in Alzheimer’s disease.

## Introduction

The formation of Aβ peptides from the amyloid precursor protein (APP) is a crucial event in Alzheimer’s disease (AD). Proteolytic processing of APP results in different forms of the Aβ protein with different characteristics [1]. C-terminal cleavage of the Aβ sequence by γ-secretase leads to the formation of Aβ1-x varying in length from 38–43 amino acids. In addition, N-terminal truncations have been identified that expose a glutamic acid at position 3 or 11. This glutamate residue can be cyclized into a pyroglutamate (pE) N-terminus which leads to the formation of Aβ_3(pE)-x_ and Aβ_11(pE)-x_ in a reaction catalyzed by glutaminyl cyclases (QCs). The pE residue stabilizes the peptide by protection against degradation by aminopeptidases [2] and lysosomal proteases [3]. In addition, the conversion of the glutamatic acid into a pE residue results in a loss of charge and the consequent increased hydrophobicity leads to an increase in aggregation propensity [4], [5]. It has been suggested that pE Aβ provides a seed for the Aβ aggregation process [6]. The change in physicochemical properties is accompanied by increased neurotoxicity of pE Aβ peptides compared to unmodified Aβ species [6]–[8].

QC is abundantly expressed in the cortex and hippocampus and its expression correlates with the appearance of pE Aβ [9], [10]. In a transgenic AD mouse model, overexpression of human QC results in an increase in Aβ_3(pE)-42_ peptides, plaque formation as well as memory impairments [11]. Moreover, the formation of the neurotoxic pE Aβ peptides can be prevented by inhibition of QC *in vitro* and *in vivo* [10], [12]. Oral application of a QC inhibitor diminishes plaque formation and improves performance in spatial learning tests and context memory in two transgenic mouse models of AD [12]. QC expression is upregulated at the mRNA and protein level in the AD brain [12], which may be an important pathogenic factor triggered early in the development of AD pathogenesis.

Accumulation of Aβ is an early pathogenic event in AD. In particular, oligomeric assemblies of Aβ interfere with cellular physiology. AD is a multifactorial disease, and additional dysfunction that is not directly related to Aβ may converge with the toxic effects of Aβ. For example, our lab has previously shown that the unfolded protein response (UPR) is activated early in AD [13], [14]. The UPR is a stress response of the endoplasmic reticulum (ER) that is activated in response to disturbed ER homeostasis. Several homeostatic and biosynthetic pathways are located in the ER: it is a site for Ca^2+^ homeostasis, redox balance, lipid synthesis and the synthesis and folding of membrane bound and secretory proteins. We showed that increased production of Aβ [15], as well as the presence of early oligomeric Aβ aggregates [16] sensitize cells for UPR induction and ER stress toxicity, indicating an interaction between these two early events in AD pathology. Recently our group demonstrated that the accumulation of pE Aβ aggregates in human cortex is highly dependent on the presence of AD pathology, but also on age [3]. Also changes in Ca^2+^ homeostasis that have been suggested to be involved early in AD pathology are highly age related [17]–[20]. The above illustrates that multiple factors can affect AD pathogenesis. In this study we investigated whether these key early pathogenic events -Aβ, UPR activation and perturbed Ca^2+^ homeostasis - affect QC expression.

## Results

### Upregulation of QC mRNA is a very Early Event in AD Pathology

**Figure 1:**
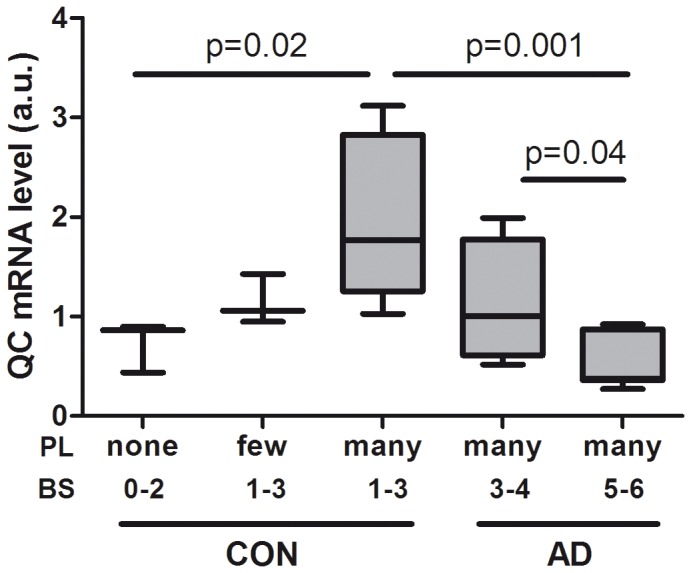
QC mRNA expression is increased in earliest stages of AD pathology. Expression of QC mRNA was determined in a cohort of patients with varying stages of AD pathology (see materials and methods for details). CON and AD refer to the clinical diagnosis, BS is Braak score for tau pathology, PL is the plaqueload in the hippocampus/entorhinal cortex. Shown is a box-plot of results of the pathological groups as indicated in hippocampus/entorhinal cortex. The expression levels were normalized to eEF2α mRNA. Kruskall-Wallis test was used to evaluate differences between groups followed by the Mann-Whitney U test, to test differences between pairs of groups.

Elaborating on the previously reported QC mRNA expression data in the neocortex of AD patients [12], we determined the expression of QC mRNA in the hippocampus/entorhinal cortex of a cohort of age-matched patients with varying stages of AD pathology. We observe a significant increase in mRNA levels of QC in the patients with the earliest stages of pathology (Fig. 1). Levels are the highest in low Braak scores for tau pathology (1–3) with many amyloid deposits in the hippocampus. In patients with more advanced tau pathology the QC levels are lower, in accordance with the observations in the cortex in the previous study. This indicates that QC is upregulated in the earliest stages of AD pathology.

### QC Expression is not Upregulated by Aβ or the UPR

**Figure 2:**
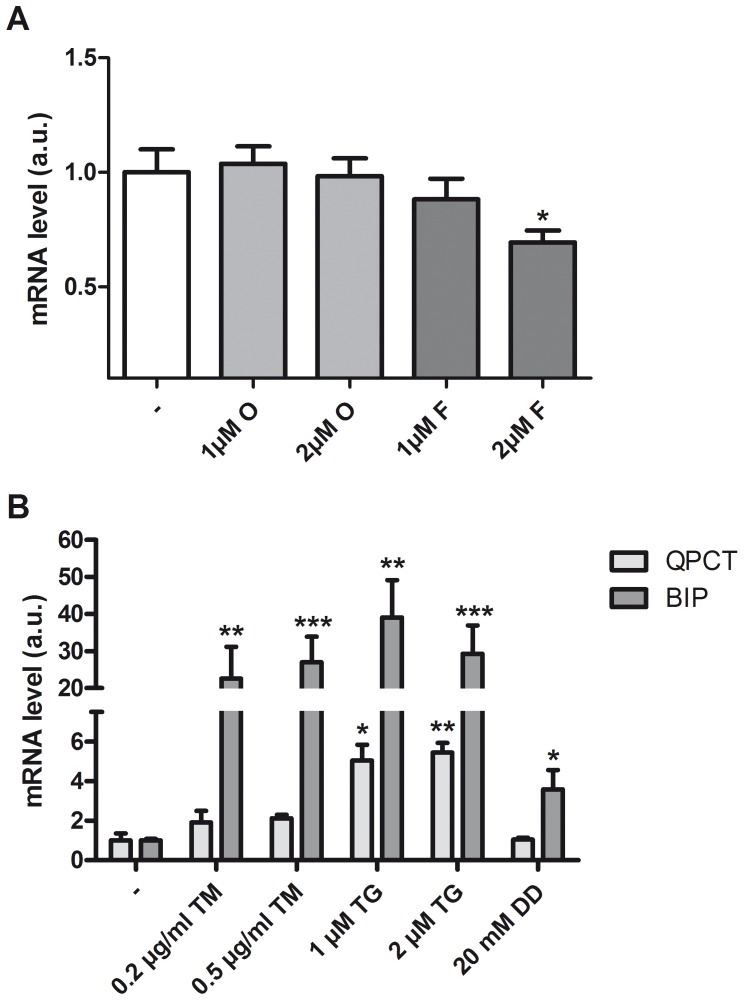
QC expression is not upregulated by Aβ or the UPR. A. Differentiated SK-N-SH cells were treated with 1 µM and 2 µM oligomeric (O) and fibrillary (F) Aβ_1–42_ for 24 h. Shown is the average + SD of normalized QC mRNA levels of triplicates from a representative experiment B. Differentiated SK-N-SH cells were treated with different UPR inducers, TM (0.2 µg/µl and 0.5 µg/µl), TG (1 µM and 2 µM) and DD (20 mM) for 16 h. Shown is the average + SD of normalized QC mRNA levels of n = 9 from 3 independent experiments. Normalized BiP mRNA levels are shown as positive control for UPR induction (n = 6 from two independent experiments). The expression levels were normalized to eEF2α mRNA, the levels in untreated cells are set to 1. Asterisks indicate a significant difference compared with control (*p≤0.01, **p≤0.001, ***p≤0.0001).

Because QC is increased very early in AD, we investigated if early events occurring in AD might be involved in the regulation. Therefore differentiated SK-N-SH were treated with increasing concentrations of either oligomeric or fibrillar Aβ_1–42_ for 24 h and subsequently the expression level of QC mRNA was measured by semi-quantitative real-time PCR analysis (Fig. 2A). Treatment with Aβ did not increase the QC mRNA level - fibrillar Aβ_1–42_ at 2 µM even slightly reduced the QC level - indicating that this is not a signal to increase QC expression.

Previously, our group demonstrated that UPR activation, which is known to affect the expression of several genes, is an early event in Alzheimer’s disease brain. Therefore we used different chemical compounds (tunicamycin [TM], thapsigargin [TG] and 2-deoxy-d-glucose [DG]), to induce the UPR in the differentiated SK-N-SH (Fig. 2B). All treatments increase the levels of the UPR-responsive mRNA BiP, confirming that the UPR is activated. In contrast, the QC mRNA expression level was not increased by TM or DG, but only after treatment with TG indicating that activation of the UPR *per se* does not regulate QC gene expression.

### Disturbed Ca^2+^ Homeostasis Increases QC Expression and Enzyme Activity

**Figure 3:**
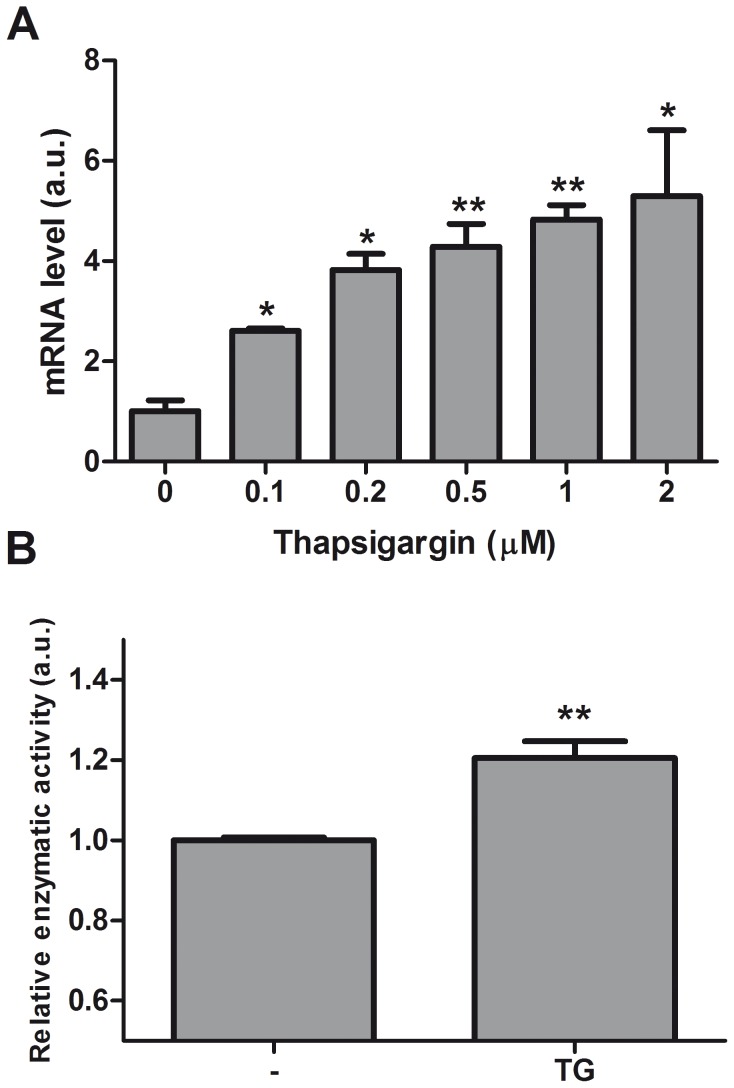
Disturbed Ca^2+^ homeostasis increases QC expression and enzyme activity. A. Differentiated SK-N-SH cells were treated with different concentrations TG as indicated. Shown are the average + SD of normalized QC mRNA levels of n = 6 from two independent experiments. The expression levels were normalized to eEF2α mRNA, the expression levels in untreated cells are set to 1. B. Differentiated SK-N-SH cells were treated with 1 µM TG for 16 h. The enzymatic activity of QC activity was determined in protein lysates as described in materials and methods, activity in untreated cells is set to 1. Shown is average +SEM of n = 15 from 5 independent experiments. Asterisks indicate a significant difference compared to control (*p≤0.001, **p≤0.0001).

To further explore the TG induced gene expression of QC, differentiated SK-N-SH cells were treated with increasing concentrations of TG. As shown in Figure 3A a dose-dependent increase in QC mRNA expression levels was observed. The TG induced expression of QC indicates Ca^2+^-dependent gene regulation since TG, a specific and irreversible sarco-endoplasmic reticulum ATPase (SERCA) pump inhibitor, blocks the re-uptake of cytosolic Ca^2+^ into the ER and as a consequence depletes the ER Ca^2+^ and increases the cytosolic Ca^2+^ as has been shown by fura-2 Ca^2+^ imaging [21]–[24].

To investigate if TG also increased QC activity, we used an enzymatic activity assay. The activity of the enzyme is determined by the ability of QC to convert a glutaminyl residue to a pyroglutamyl residue on a fluorogenic substrate. Differentiated SK-N-SH cells were treated with 1 µM TG for 16 h. The fluorescence, indicative of the activity of QC, was measured and the data show that treatment with TG induces the fluorescence which is in line with the mRNA expression data (Fig. 3B).

### ER Ca^2+^ Depletion Increases QC Levels

**Figure 4:**
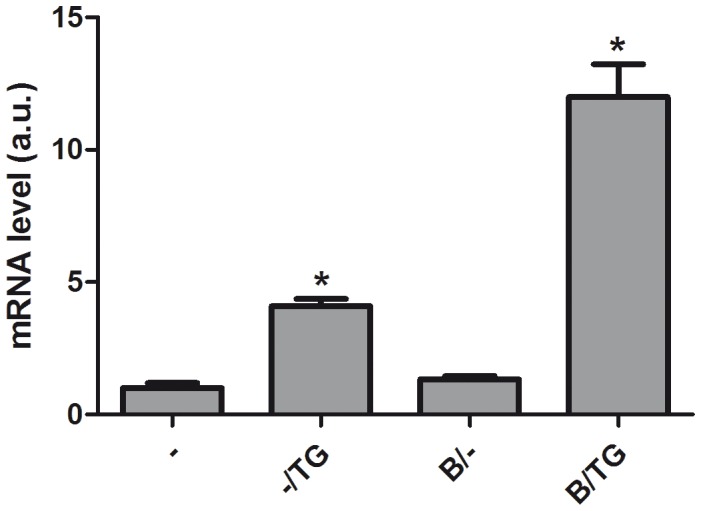
ER Ca^2+^ depletion increases QC levels. Differentiated SK-N-SH cells were treated with 1 µM TG for 16 h with or without pre-incubation with 5 µM BAPTA-AM (B) for 1 h. Shown are the average + SD of normalized QC mRNA levels of n = 9 from 3 independent experiments. The expression levels were normalized to eEF2α mRNA, the expression levels in untreated cells are set to 1. Asterisks indicate a significant difference compared to control (*p≤0.0001).

Conceivably two TG-dependent events could regulate QC mRNA levels (i) TG-mediated ER Ca^2+^ pool depletion and/or (ii) TG-initiated rise in cytosolic Ca^2+^. To investigate which of these two mechanism might be responsible for the TG initiated increase in QC mRNA levels, differentiated SK-N-SH were pretreated (1 h) with the membrane permeable Ca^2+^ chelator BAPTA-AM (5 µM) before treatment with TG (16 h) (Fig. 4). Once BAPTA-AM enters the cytosol, it is cleaved to membrane-impermeable BAPTA and significantly reduces and maintains a low cytoplasmic Ca^2+^ concentration even upon subsequent stimulation with TG [25], [26]. When BAPTA-AM was added separately no increase in QC mRNA expression level was observed. However, the combination of BAPTA and TG strongly potentiated the TG induced QC mRNA expression level (Fig. 4), indicating that it is probably not the rise in the cytosolic Ca^2+^ but rather the depletion of the ER Ca^2+^ that is responsible for the TG induced QC gene expression.

### Induction of c-fos and c-jun Precedes Increased QC Expression

**Figure 5:**
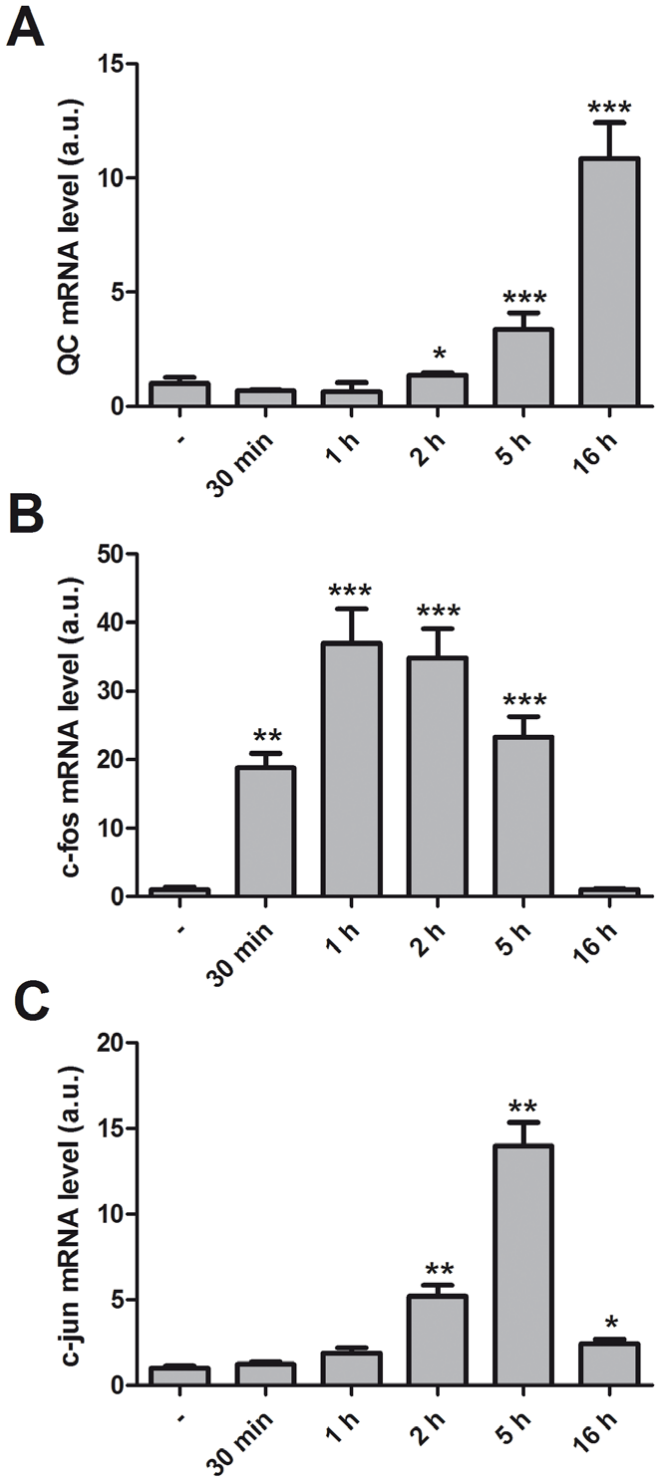
Induction of c-fos and c-jun precedes increased QC expression. Differentiated SK-N-SH cells were treated with 1 µM TG for the times indicated. Shown is the average + SD of normalized QC (A), c-fos (B) and c-jun (C) mRNA levels of triplicates from a representative experiment of three. The expression levels were normalized to eEF2α mRNA, the expression levels in untreated cells are set to 1. Asterisks indicate a significant difference compared to control (*p<0.05; **p≤0.001; ***p≤0.0001).

To further analyze the effect of TG on QC expression we determined the expression of QC at different time points (Fig. 5A). The earliest timepoint where a slight increase is observed is 5 h, but the levels are further increased at 16h. This relatively slow effect indicates an indirect mechanism, and can be explained if the effect is mediated via the upregulation of a Ca^2+^ -dependent transcription factor.

**Figure 6:**
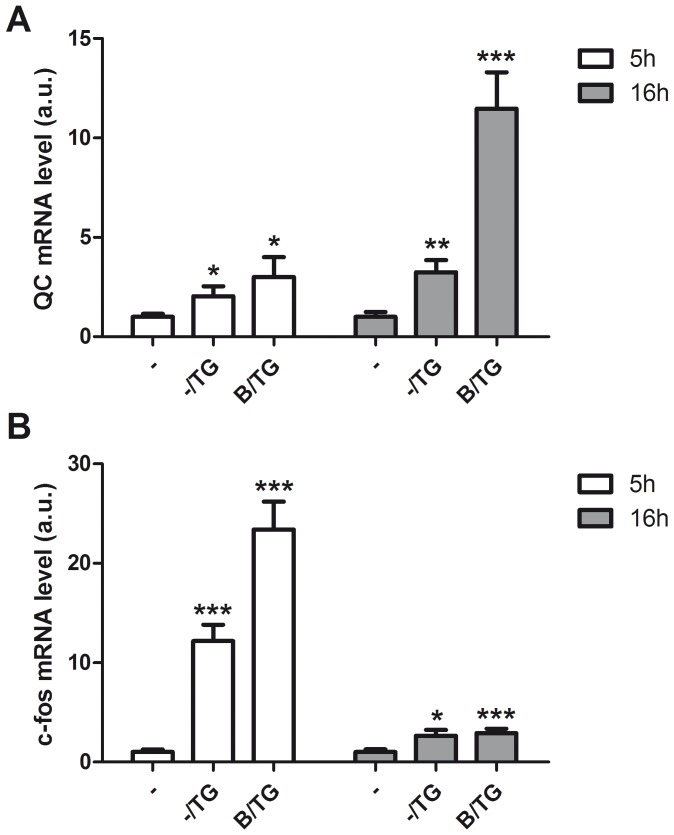
Induction of c-fos by ER Ca^2+^ depletion precedes QC expression. Differentiated SK-N-SH cells were treated with 1 µM TG for 5 h and 16 h with or without pre-incubation with 5 µM BAPTA-AM (B) for 1 h. Shown are the average + SD of triplicates of normalized QC (A) and c-fos (B) mRNA levels from a representative experiment of three. The expression levels were normalized to eEF2α mRNA, the expression levels in untreated cells are set to 1. Asterisks indicate a significant difference compared to control (*p<0.05, **p≤0.02 ***p≤0.001).

We used Tfsearch (www.cbrc.jp/research/db/TFSEARCH.html) to identify possible Ca^2+^ -dependent transcription factor binding sites in the proximal promoter region of the *QC* gene. The program identified a putative binding site for heterodimers of the transcription factors encoded by the immediate early genes (IEGs) c-fos and c-jun. Disturbed ER Ca^2+^ homeostasis in neurons leads to the rapid activation of c-fos and c-jun [27]. Therefore we investigated the effect of TG on c-fos and c-jun expression. We found that both IEGs are rapidly induced by TG, c-fos faster than c-jun, but both are high at 5 h after treatment, whereas after 16 h, both are back to their normal levels (Fig. 5B,C). Like QC, the expression of c-fos by TG is also potentiated by treatment with BAPTA-AM. Again, this effect is observed at 5 h for c-fos, and at 16 h for QC (Fig. 6). These data demonstrate that c-fos and c-jun expression precedes QC expression and suggest that the upregulation of QC may involve activation of the QC promoter via c-fos and c-jun.

### Ca^2+^ Induced QC and c-fos mRNA Expression is Neuronal Cell Type Specific

**Figure 7:**
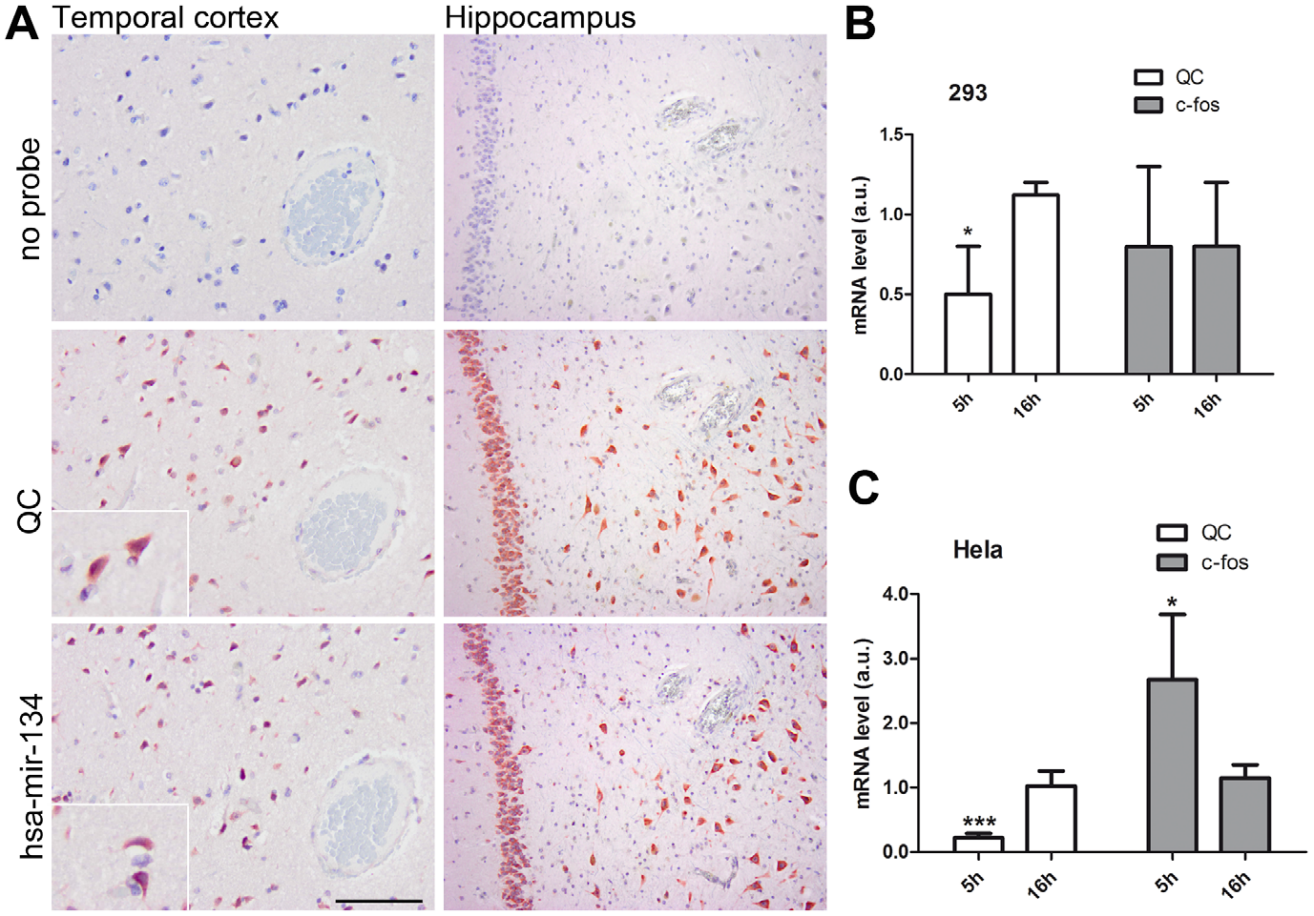
Ca^2+^ induced QC and c-fos mRNA expression is neuronal cell type specific. A. *In situ* hybridizations were performed on sequential sections of temporal cortex and hippocampus/entorhinal cortex of a patient with early AD pathology (I,B) as described in materials and methods. Shown are incubations without probe, using a probe directed against the QC mRNA or a probe recognizing the neuron-specific microRNA hsa-mir-134. Scale bar: 100 µM. B. Hek293 and C. Hela cells were treated with 1 µM TG for 5 h and 16 h. Shown is the average + SD of normalized QC and c-fos mRNA levels of n = 6 from 2 experiments (B) and n = 12 from 4 experiments (C). The expression levels were normalized to eEF2α mRNA, the expression levels in untreated cells are set to 1. Asterisks indicate a significant difference compared to control (*p<0.05, ***p≤0.0001).

Since Aβ is known to be produced by different cell types in the brain, we investigated which cells express QC. We employed *in situ* hybridization using LNA probes, to detect the QC mRNA in human temporal cortex and hippocampus/entorhinal cortex. In sequential slides, probes recognizing the QC mRNA and the neuron specific microRNA hsa-mir-134 were used. This demonstrated that QC is predominantly expressed in neurons in different brain areas (Fig. 7A).

To analyze whether the Ca^2+^-dependent regulation of QC is cell-type specific, we treated the non-neuronal HEK293 and Hela cell lines with TG. QC is not upregulated by TG treatment in either cell line (Fig. 7B,C). At 5h, the QC expression is even significantly reduced, but at 16 h there is no effect of TG observed. Interestingly, also the induction of c-fos by TG appears to be cell type specific. In the Hela cells the response is much lower than in the SK-N-SH cells and in 293 cells TG does not elicit any effect on the c-fos levels (Fig. 7B,C). These data suggest that the Ca^2+^-dependent regulation of QC expression is specific for neuronal cells.

## Discussion

The expression of QC, the enzyme that catalyzes the conversion of Aβ to the more toxic pE Aβ, is upregulated in AD cortex. Here we demonstrate that also in hippocampus/entorhinal cortex the upregulation of QC occurs at the earliest stages of AD pathology. In this study we investigated whether early pathogenic events that occur in AD brain could be directly involved in this regulation. We demonstrated that QC mRNA expression was not increased by oligomeric or fibrillar Aβ aggregates. Also induction of the UPR, using TM and 2DG was without effect. Only when the UPR was induced by the SERCA pump inhibitor TG the QC mRNA level was increased, indicating the involvement of Ca^2+^. We tried three different commercially available QC antibodies to directly determine the QC protein levels, but none worked in our hands. Instead we demonstrated that the QC enzyme activity is increased by TG. Aβ has been shown before to affect Ca^2+^ homeostasis [28], but apparently in a different manner or to a lesser extent than the Ca^2+^ perturbing stimuli that induce QC expression. Ca^2+^ signalling plays an important role in the regulation of many processes in cells. In neurons, Ca^2+^ acts as a second messenger and co-factor for many processes, e.g. neurotransmitter release and long-term potentiation, the cellular mechanism underlying learning and memory [29]. Since Ca^2+^ is involved in such a broad variety of processes, the Ca^2+^ signals need to be regulated precisely and this involves regulated changes in Ca^2+^ concentration in the different cellular compartments. Under resting conditions the cytosolic Ca^2+^ concentration is kept low by an array of buffers, pumps and transport mechanisms, creating an electro-chemical gradient across the plasma membrane and the intracellular Ca^2+^ stores, including the ER. Ca^2+^ is predominantly released from the ER through activation of inositol-1,4,5-triphosphate receptors (IP_3_Rs) and ryanodine receptors (RyRs), whereas the SERCA pumps refill the store after depletion [30]. Inhibition of SERCA leads to an increase in cytosolic Ca^2+^ concentration and ER Ca^2+^ depletion. Our data show that chelation of intracellular Ca^2+^ using BAPTA-AM potentiates the QC induction by TG, but does not have an effect by itself. Combined use of TG and BAPTA-AM results in faster and increased release of Ca^2+^ from the ER. This indicates that depletion of ER Ca^2+^ stores is the main trigger to increase the expression of QC. To further define the mechanism we analysed the effect of SERCA inhibition in combinations with pharmacological inhibition of the IP_3_Rs and RyRs. Unfortunately these experiments were not informative, probably by artefacts generated by the use of combinations of different pharmacological inhibitors. In addition, it has been shown before that the ER Ca^2+^ leak is not affected by inhibition of the IP_3_Rs or of the RyRs [31], probably because of the presence of other Ca^2+^ leak mediators [32].

Activation of immediate early gene expression is a key event in stress-induced neuronal cell injury [33]. Analysis of the *QC* promoter showed the presence of a potential binding site for the heterodimer of the transcription factors c-fos and c-jun, both IEGs. We found that c-fos and c-jun mRNA are induced by the same stimuli as QC mRNA. It was reported before that c-fos is induced in neurons by treatment with TG [27]. Similar to what we report in this study for QC, this increase is caused by Ca^2+^ depletion of the ER, rather than increased cytosolic Ca^2+^ levels. The induction of the IEGs is much faster and precedes the upregulation of QC. This is compatible with a model where the upregulation of QC by ER Ca^2+^ depletion is mediated by c-fos and c-jun, although this requires further study. Interestingly, both c-fos and c-jun are upregulated in AD hippocampus [34].

Using *in situ* hybridization we demonstrated that QC is predominantly expressed in neurons in the human brain, both in temporal cortex as well as in the earlier affected area in AD, the hippocampus/entorhinal cortex. The regulation of QC expression by TG appears to be specific for neuronal cells, because it is not observed in non-neuronal cells. Also c-fos is less responsive in non-neuronal cells, which strengthens the hypothesis that IEG induction is required for Ca^2+^ -regulated QC expression. BiP is upregulated in response to TG treatment in these cells, indicating that the treatment has resulted in depletion of ER Ca^2+^ levels. It is possible that non-neuronal cells have more resources to deal with disturbances in Ca^2+^ homeostasis, or that neuronal cells are more dependent on proper Ca^2+^ homeostasis for their function. It has been shown before that a different isoform of QC, isoQC, is involved in monocyte infiltration via modification of cytokines, and therefore it has a major role in cell types involved in the immune response [35]. Our data suggest that QC performs a specific function in neurons. The formation of an N-terminal pE residue is an important event in the processing of numerous bioactive neuropeptides, hormones and cytokines during their maturation in the secretory pathway [36], [37]. These peptides need the conformational change to bind to their receptors and/or to protect the N terminus from degradation. It is possible that QC is upregulated under stress conditions in neurons to stabilise specific peptides and potentiate their function. Whether the modification of Aβ is part of this or whether this is a pathological side-effect requires further investigation.

Perturbed Ca^2+^ homeostasis has been found in both sporadic and in familial (F)AD [38], [39]. The majority of cases of FAD originate from a mutant PS gene. In addition to effects on Aβ formation, PS-FAD mutations lead to perturbed neuronal ER Ca^2+^ signalling [40], [41]. It was found that PS can function as low-conductance passive ER Ca^2+^ leak channel and that the FAD mutations disrupt this Ca^2+^ leak function [42]. This results in higher steady-state intraluminal Ca^2+^ levels and lower cytosolic Ca^2+^ homeostasis levels and leads to exaggerated release of Ca^2+^ from the ER, both in *in vitro* and *in vivo* [43], [44]. In this respect it is interesting that the intraneuronal levels of pE Aβ are higher in PS1 FAD patients than in sporadic AD patients [45]. In sporadic AD, aging is the major risk factor for developing the neurodegenerative disease. The Ca^2+^ hypothesis of aging emerged two decades ago [18] and is supported by studies that show age-related alterations in specific Ca^2+^ homeostasis -regulating systems in neurons [19], [20]. In the aged brain there is an increased Ca^2+^ release from the ER stores through both the RyR and the IP_3_ receptors [46]. Interestingly, our group recently demonstrated that the accumulation of intracellular pE aggregates in human brain is also highly dependent on age [3]. The data presented here suggest that perturbed ER Ca^2+^ homeostasis facilitates the production of pE modified Aβ and may thereby contribute to the amyloid pathology in AD.

## Materials and Methods

### Cell Culture and Treatment

SK-N-SH (European Collection of Cell Cultures #86012802, Salisbury, UK), 293 and Hela cells were cultured in Dulbecco’s modified Eagle’s medium with GlutaMAX (Gibco BRL, Carlsbad, CA, USA) supplemented with 10% fetal calf serum (FCS, Gibco BRL), 100 µg/ml streptomycin and 100 U/ml penicillin. SK-N-SH cells were differentiated in culture medium supplemented with all *trans*-retinoic acid (Sigma, St Louis, MO, USA) at a final concentration of 10 µM for 5 days. Differentiated cells were treated with Aβ_1–42_ oligomers or fibrils, TG, TM, DG (all from Sigma) and BAPTA-AM (Calbiochem, Darmstadt, Germany) at the indicated concentrations and time points.

### Preparation of Different Aβ Species

Synthetic Aβ_1–42_ was purchased from Anaspec (San Jose, CA, USA). Aβ_1–42_ was dissolved in hexafluorisopropanol (HFIP) at a final concentration of 1 mg/ml, aliquoted and dried under vacuum. The resulting peptide film was stored at −80°C until further use. To obtain low molecular weight Aβ, the peptide film was dissolved in DMSO (5 mM), and sonicated in a bath sonicator for 10 minutes. Aβ was subsequently diluted in PBS to a final concentration of 50 µM. To enrich for oligomers, this mixture was incubated at 4°C for 24 h. High order aggregates were removed by centrifugation (10 min, 14 000 rpm) at 4°C. To obtain fibrillar Aβ, the peptide film was dissolved in DMSO (5 mM). Then 10 mM HCl was added to bring the peptide to 100 µM concentration and incubated for 24h at 37°C. The final concentration of the oligomeric and the fibrillar Aβ 1–42 preparations was determined using Bradford protein assay and the β-sheet content was determined by a Thioflavin T assay.

### Post-mortem Brain Tissue

Post-mortem brain material was obtained from the Netherlands Brain Bank (Amsterdam, The Netherlands). All donors or their next of kin provided written informed consent for brain autopsy and use of tissue and medical records for research purposes. The parameters of all tissue samples used in this study are listed in Table 1.

**Table 1:** Post-mortem brain material used in this study.

Case	Clinical diagnosis	Braak stage	Gender	Age	ApoE genotype	PMI
**1**	CON	0	M	80	33	07∶15
**2**	CON	2	F	93	33	05∶50
**3**	CON	1	M	84	33	07∶05
**4**	CON	2	M	87	33	07∶20
**5**	CON	1	F	83	32	05∶30
**6**	CON	1	F	81	33	06∶40
**7**	CON	1	M	85	44	04∶15
**8**	CON	2	F	86	43	06∶25
**9**	CON	1	M	91	33	08∶00
**10**	CON	1	F	85	33	05∶00
**11**	AD	4	F	94	33	05∶00
**12**	CON	3	F	91	32	05∶20
**13**	AD	3	M	86	43	05∶35
**14**	CON	3	M	74	43	05∶00
**15**	AD	4	F	86	43	05∶55
**16**	AD	4	F	84	43	04∶50
**17**	AD	5	F	89	33	10∶20
**18**	AD	6	F	81	33	06∶00
**19**	AD	6	M	69	43	05∶00
**20**	AD	6	F	67	32	06∶05
**21**	AD	5	M	75	43	05∶25
**22**	AD	5	F	94	33	04∶30
**23**	AD	6	F	91	43	05∶45

Listed are clinical diagnosis (CON = control, AD = Alzheimer’s disease), Braak stage, gender, age (in years), ApoE genotype, post-mortem interval (PMI, in hours: minutes).

### RNA Isolation and Real-time Quantitative PCR

Total RNA was isolated from differentiated SK-N-SH cells, untreated or treated, and post-mortem brain tissue with the RNAeasy minikit (Qiagen, Netherlands). cDNA synthesis was performed using Superscript II reverse transcriptase (Invitrogen, Carlsbad, CA) and oligo-dT primers. Real-time quantitative PCR (qPCR) was performed using the Light Cycler 480 system (Roche Applied Science, USA). Reaction volumes of 5 µl contained cDNA, 0.1 µM Universal Probe Library probe (Roche Applied Science, USA), 0.4 µM forward primer, 0.4 µM reverse primer and 2.5 µl 2x LightCycler 480 Probes Master (Roche Applied Science, USA). After denaturation for 10 min at 95°C, amplification was performed using 35 cycles of denaturation (95°C for 10 s), followed by annealing (58°C for 15 s), and elongation (72°C for 15 s). Results were analysed using the LightCycler 480 software (Roche Applied Science, USA) version 1.5. Expression levels were normalized using eEF2α. Primers and probes used in this study are listed in Table 2.

**Table 2:** Primers and probes used for qPCR 480 light cycler.

Gene	Primers	Product size (bp)	Probe no.
QC	Fw: TGC AAA GAT GGC ATC GACRev: CCA ATC AAA TCC AAT AAG ACC AA	93	#1
c-Fos	Fw: CTA CCA CTC ACC CGC AGA CTRev: AGG TCC GTG CAG AAG TCC T	72	#67
c-Jun	Fw: CCA AAG GAT AGT GCG ATG TTTRev: CTG TCC CTC TCC ACT GCA AC	62	#19
BiP	Fw: GCT GGC CTA AAT GTT ATG AGG ARev: CCA CCC AGG TCA AAC ACC	110	#7
Eef2α	Fw: CAA TGG CAA AAT CTC ACT GCRev: AAC CTC ATC TCT ATT AAA AAC ACC AAA	122	#63

Probe numbers refer to numbers in the Roche universal probe library.

### Activity Assay

Cells were washed with PBS and lysed in PBS+1%Triton. Subsequently the supernatant was collected after centrifugation 12000×g at 4°C for 10 minutes. The protein concentration was determined by the Bradford Assay (Bio-Rad, Laboratories, Veenendaal, The Netherlands). 100 µl lysate (0.5 mg/ml) was mixed with 100 µl of 200 µM Q-AMC (Bachem, The Netherlands) in assay buffer (25 mM HEPES, pH 7.0) and incubated in dark tubes at room temperature for 20 minutes. The reaction was stopped at 100°C for 5 minutes and the incubations were cooled on ice for 3 minutes. Subsequently 200 µl of rhPGPEP-1 (0.1 µg/ml, 6278-CY, R&D systems, Europe) was added to each vial and the mixture was incubated at room temperature for 10 minutes. Fluorescence was measured using a FLUOstar Omega (BMG LABTECH GmbH, Ortenberg, Germany).

### In situ Hybridization

In situ hybridization was essentially performed as described before [47]. For human QC a 5′ fluorescein labeled 19mer antisense oligonucleotide was used, containing Locked Nucleic Acid (LNA) and 2′OME RNA moieties (FAM – AuuTucTucAuuGucAucC, capitals indicate LNA, lower case indicates 2′OME RNA). An antisense oligo targeting the neuron-specific hsa-miR-134 was used (FAM - CucTggTcaAccAguCacA) to detect neurons and as negative control an oligo that does not detect a signal in human brain (FAM - TaaCccTaaGgcAauTccT). The oligonucleotides were synthesized by Ribotask ApS, Odense, Denmark. The hybridizations were done on 5 µM sections of paraffin embedded temporal cortex material obtained from the Netherland Brain Bank. In brief, sections were deparaffinized, treated with proteinase K (20 µg/ml) for 5 min. Hybridizations were done at 60°C for 90 min in hybridization mix (50% (vol/vol) deionized formamide, 600 mM NaCl, 10 mM HEPES buffer, pH 7.5, 1 mM EDTA, 5×Denhardt’s reagent and 200 µg/ml denatured herring sperm DNA). The oligonucleotide concentration in the hybridization mix was 1 µM. The QC incubations were also performed at lower concentration (0.25 µM) with similar result. After hybridization the tissue sections were washed consecutively for 5 min with 2×SSC, 0.5×SSC and 0.2×SSC at 60°C. The hybridization signal was detected using a rabbit polyclonal α-fluorescein/Oregon green antibody (Molecular Probes) and a horse radish peroxidase (HRP) labeled goat α-rabbit polyclonal antibody as secondary antibody. Signal was detected with standard 3-amino-9-ethylcarbazole staining (AEC) and hematoxylin was used as a nuclear counterstain.
